# Probable involvement of p11 with interferon alpha induced depression

**DOI:** 10.1038/srep17029

**Published:** 2016-01-29

**Authors:** Jiqiang Guo, Wen Zhang, Lili Zhang, Huaxia Ding, Jingjing Zhang, Chen Song, Yanfei Zhang, Namei Xia, Mingfang Li, Yinming Liang, Xianzhang Hu, Haojiang Luan, Hui Wang

**Affiliations:** 1 Research Center for Immunology, School of Basic Medical Sciences, Xinxiang Medical University, China; 2 Collaborative Innovation Center of Molecular Diagnosis and Laboratory Medicine in Henan Province, School of Laboratory Medicine, Xinxiang Medical University, China; 3 Laboratory of Molecular Biology, National Institute of Mental Health, USA

## Abstract

Depression is one of the major side effects of interferon alpha (IFN-α) treatment, but the molecular mechanism underlying IFN-α-induced depression remains unclear. Several studies have shown that the serotonin receptors 5-HTR1b and 5-HTR4 play key roles in the anti-depression effects associated with p11 (S100A10). We investigated the effects of IFN-α on the regulation of p11, 5-HTR1b and 5-HTR4 in mice and human neuroblastoma cells (SH-sy5y). We found that intraperitoneal injection with IFN-α in Balb/c mice resulted in an increased immobility in FST and TST, and potently lowered the protein levels of p11, 5-HTR1b and 5-HTR4 in the hippocampus or cingulate gyrus. IFN-α significantly down-regulated the protein levels of p11, 5-HTR1b and 5-HTR4 in SH-sy5y cells, in a time- and dose-dependent manner. Our study revealed that over-expression of p11 could prevent the IFN-α-induced down-regulation of 5-HTR1b and 5-HTR4. The results indicated that IFN-α treatment resulted in p11 down-regulation, which subsequently decreased 5-HTR1b and 5-HTR4 *in vitro* or *in vivo*. Our findings suggested that p11 might be a potential regulator on 5-HTR1b and 5-HTR4 as well as a predictor of or a therapeutic target for IFN-α-induced depression.

Interferon-α (IFN-α) is one of the classic innate immune cytokines widely used in antiviral^1^,^2^ and antitumor therapies^3^. However, IFN-α often causes psychiatric side-effects, such as depression, fatigue, insomnia, anxiety, and cognitive disturbances in a long-term treatment^4^. It is estimated that approximately 30–70% of hepatitis C virus-infected patients treated with IFN-α experience different degrees of depression^5^. Such depression can lead to interruption of IFN-α treatment in patients^6^. It is of great clinical importance to investigate the mechanism underlying IFN-α-induced depression.

Results from the studies on molecular mechanism underlying IFN-α-induced depression remain inconsistent. It is suggested that pro-inflammatory cytokines, including IL-1, TNF-α and IL-6 are involved in the onset of depression, for the pro-inflammatory cytokines can affect the peripheral nerves and also directly or indirectly affect the central nervous system to induce mental disorders^7^,^8^,^9^,^10^,^11^. Others find that IL-1β is undetectable in human plasma under normal physiological or pathological conditions, and the fluctuations of IL-6 and TNF-α are very poorly discriminated in non-depressive and depressive patients^12^. One hypothesis is that IFN-α can significantly disturb the metabolism of tryptophan via increasing the expression of indoleamine 2, 3-dioxygenase (IOD), and subsequently inhibit the synthesis of 5-hydroxytryptamine (5-HT), which ultimately leads to neurotransmitter conduction disorders in the synapses^13^,^14^. It is also reported that the alterations of tryptophan metabolism have no relationship with psychiatric side effects in cancer patients treated with IFN-α^15^. In addition, there is no association between the variants in the IOD gene polymorphisms and the diagnosis of the IFN-α-induced depression^16^.

p11, or S100A10, a member of the S100 family of proteins with 2 EF-hand calcium-binding motifs, is recently reported to play a crucial role in the onset of depression by interacting with the 5-hydroxytryptamine receptor 1b (5-HTR1b) or 5-hydroxytryptamine receptor 4 (5-HTR4)^17^,^18^. The levels of the p11 protein are significantly increased in some rat brain regions after treatment with anti-depressants^19^, whereas p11 mRNA levels are significantly decreased in the depressive rodent model and in patients with severe depression^20^,^21^. p11 knockout mice show spontaneous depressive behavior and a low response to 5-HT1b-stimulating agents^17^,^22^,^23^. However, it remains unknown whether p11 is involved in IFN-α-induced depression.

5-HTR1b and 5-HTR4 are reportedly critical receptors involving in the development of depression. 5-HTR1b is previously identified to be an autoreceptor that inhibits the release of serotonin in the presynaptic membrane^24^. Recently, the activation of 5-HTR1b in the post-synapse was found to facilitate excitatory synaptic transmissions associated with depression^25^. 5-HTR1b agonists are found to be effective anti-depressants in animal models^26^. 5-HTR4 plays roles in both the peripheral and central nervous systems to modulate the release of various neurotransmitters^27^. 5-HTR4 agonists have rapid effects on depressive behaviors, and its activation is necessary for these effects of serotonin-specific reuptake inhibitors (SSRIs)^28^. 5-HTR1b and 5-HTR4 have become novel targets for the pathophysiology of depression and for antidepressants therapies^29^,^30^. Even though the anti-depression effects of p11 have been well documented and an uncovered fundamental mechanism suggests that p11 alters the distribution of 5-HTR1b and 5-HTR4 in cells^17^,^18^, the effect of p11 on the expression of 5-HTR1b and 5-HTR4 is still unknown. Furthermore, the regulating effects of IFN-α on levels of 5-HTR1b and 5-HTR4, and its dependence on p11 still need to be elucidated.

In the present study, we hypothesized that IFN-α treatment resulted in depression through the inhibition of p11 and subsequent reduction of the density of 5-HTR1b/4 in nerve synapses of brain’s certain areas which were associated with depression, thereby causing nervous system dysfunction and, ultimately, depression. Therefore, we investigated the behavioral and histological responses of Balb/c mice after IFN-α treatment and the relationship between p11 and 5-HTR1b/4 in a human neuroblastoma cell line. Our study revealed that IFN-α treatment decreased p11 protein expression. 5-HTR1b and 5-HTR4 were also significantly declined after IFN-α treatment. Over-expression of p11 is sufficient to prevent the IFN-α-induced down-regulation of 5-HTR1b and 5-HTR4.

## Results

### IFN-α treated mice showed increased immobility in force swimming tests (FST) and tail suspension tests (TST)

**Figure 1:**
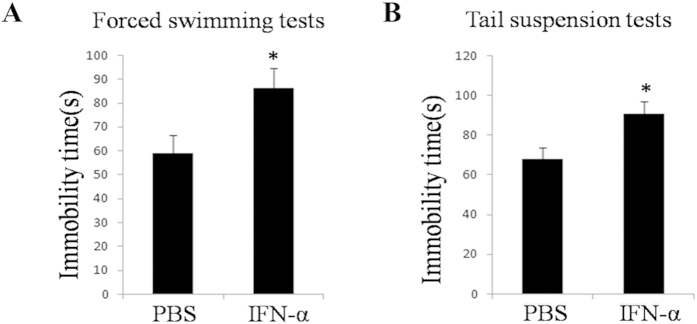
Effects of hIFN-α-2b on the immobility of mice in FST and TST. The tests were performed after the wide-type male Balb/c mice had received 10 daily intraperitoneal injections of 1000 IU/mL hIFN-α-2b (n = 15), and equal volumes of PBS as control (n = 15). (**A**) Immobility scores in FST during 2-6 min. (**B**) Immobility scores in TST during 2-6 min. The data represented the mean ± S.E. compared with the controls. *P < 0.05.

To evaluate the behavioral consequences of IFN-α injection, wild-type male Balb/c mice were intraperitoneally injected with Human recombinant IFN-α-2b (hIFN-α-2b) (n = 15) or Phosphate Buffer Solution (PBS) (n = 15) for 10 days, and then depression-like behavioral profiles of the mice were assayed using FST and TST. The hIFN-α-2b treated mice exhibited increased immobility in FST (*P* = *0.0235, F* = *1.32*) and in TST (*P* = *0.0123, F* = *1.105*), compared with the controls (Fig. 1).

### IFN-α down-regulated the protein levels of p11, 5-HTR1b and 5-HTR4 in the hippocampus and cingulate gyrus of mice

**Figure 2:**
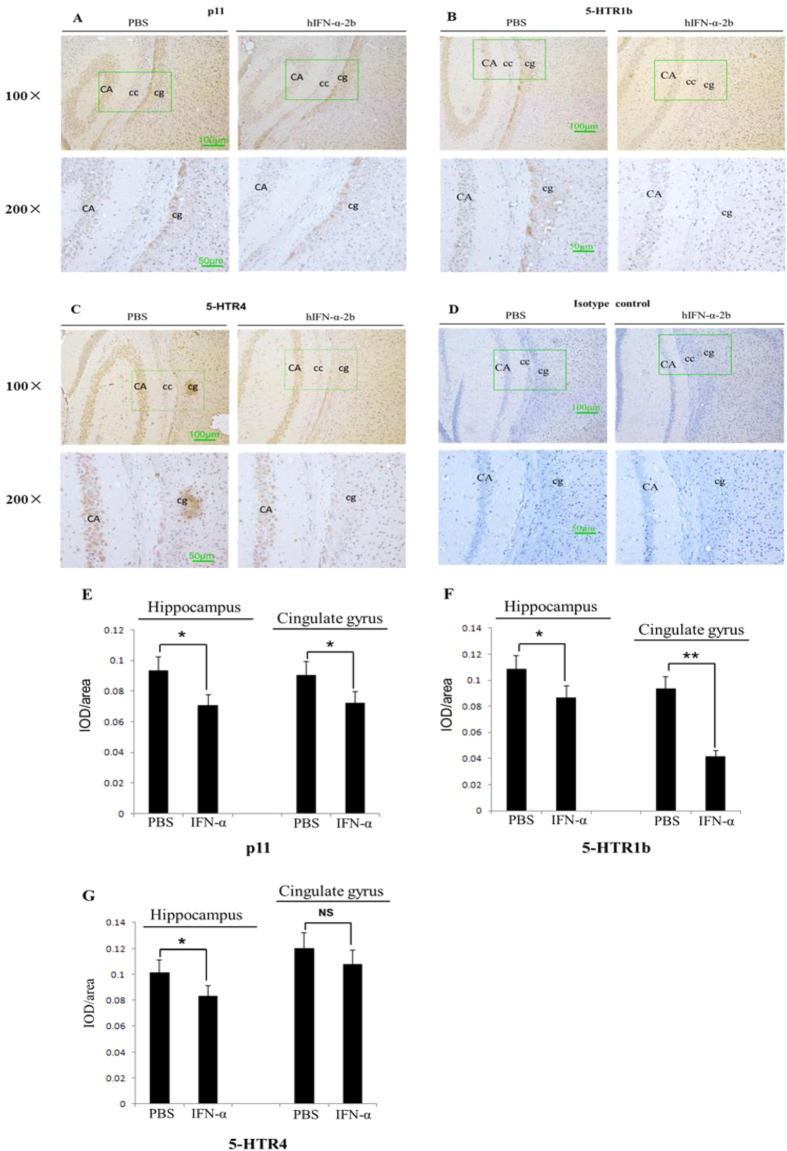
Immunohistochemistry tests assayed the p11, 5-HTR1b and 5-HTR4 protein levels in the hippocampus or cingulate gyrus in hIFN-α-2b (or PBS)-treated Balb/c mouse. (**A–C**) Maps of the brain tissues showed the protein levels in the hippocampus (CA) or cingulate gyrus (cg), where cc represented the corpus callosum. (**D**) Isotype control for immunohistochemistry using rabbit IgG instead of the primary antibody. Each image comprised four micrographs (**A–D**), where left side of the image showed the PBS treatment group and right side of the image showed the hIFN-α-2b treatment group; upstream with 100× visual fields and downstream with 200× visual fields, which corresponded to the areas in the 100× micrographs marked by green-line boxes. (**E–G**) Integrated optical density (IOD) in the positive areas of the mouse hippocampus or cingulate gyrus, which were measured using “Image Improplus 6.0” software. The IOD/area values were shown in the histogram. (**E**) hIFN-α-2b treatment reduced the p11 protein levels in the hippocampus and cingulate gyrus compared with the controls. (**F**) hIFN-α-2b treatment decreased the 5-HTR1b protein levels in the hippocampus and cingulate gyrus compared with controls. (**G**) hIFN-α-2b treatment decreased the 5-HTR4 protein levels in the hippocampus but not in the cingulate gyrus compared with the controls. Each group, n = 6; scale bar = 100 μm in 100× micrographs, scale bar = 50 μm in 200× micrographs. The data represented the mean ± S.E. compared with the controls. *P < 0.05 and **P < 0.01, and *NS*, no significant difference.

To explore the effects of IFN-α on the protein levels of p11, 5-HTR1b and 5-HTR4 in the mice hippocampus and cingulate gyrus, Human recombinant IFN-α-2b (hIFN-α-2b) was intraperitoneally injected into Balb/c mice (1000 IU/g/day) for 10 days. The protein levels in the hippocampus and cingulate gyrus were immunohistochemically analyzed. Compared with the protein-positive integrated optical density (IOD)/area in the hippocampus and cingulate gyrus of the control, the protein levels were significantly reduced in hIFN-α-2b treated mice by 24.3% (*P* = *0.0121, F* = *1.577*) and 19.9% (*P* = 0.0426, *F* = *1.22*) for p11 (Fig. 2A,E), 19.9% (*P* = *0.0361, F* = *2.137*) and 55.7% (*P* = *0.0003, F* = *1.888*) for 5-HTR1b (Fig. 2B,F) and 17.8% (*P* = *0.024, F* = *1.856*) and 10.4% (*P* = *0.2595, F* = *1.013*) for 5-HTR4 (Fig. 2C,G), respectively.

**Figure 3:**
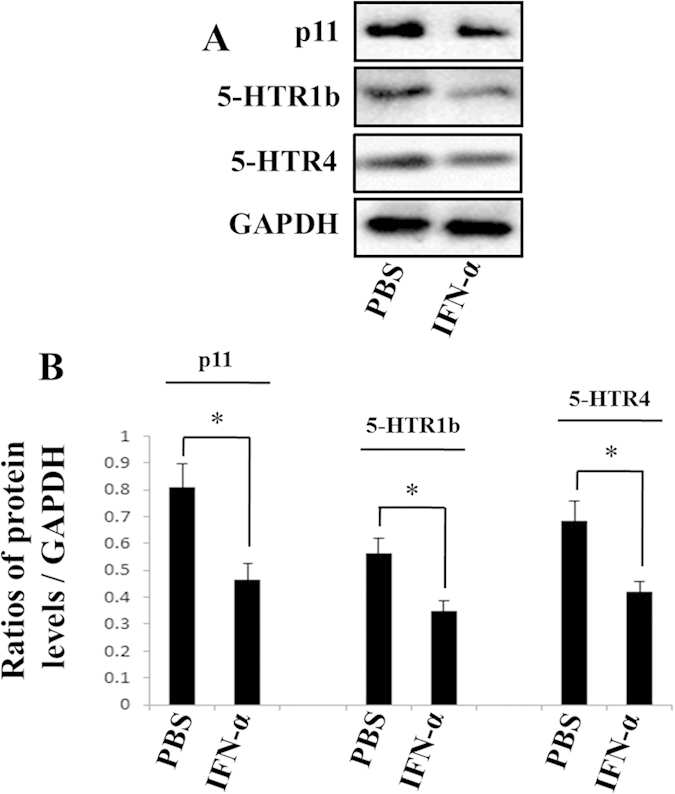
Western blots tests assayed the p11, 5-HTR1b and 5-HTR4 protein levels in the hippocampus of hIFN-α-2b (or PBS)-treated Balb/c mouse. (**A**) IFN-α treatment reduced the p11, 5-HTR1b and 5-HTR4 protein levels in the hippocampus. (**B**) The protein levels were normalized against GAPDH. Each group, n = 5. The data represented the mean ± S.E. compared with the controls. *P < 0.05.

To confirm the specificity of primary antibodies used in immunohistochemistry (IHC) tests, the proteins of mice hippocampus were assayed using western blots. The protein levels were significantly reduced in hIFN-α-2b treated mice by 42.16% (*P* = *0.0122, F* = *2.331*) for p11, 37.81% (*P* = *0.0116, F* = *2.733*) for 5-HTR1b and 38.23% (*P* = *0.0130, F* = *3.777*) for 5-HTR4, respectively (Fig. 3A,B).

### IFN-α dramatically reduced the p11 protein levels in a time and dose-dependent manner

**Figure 4:**
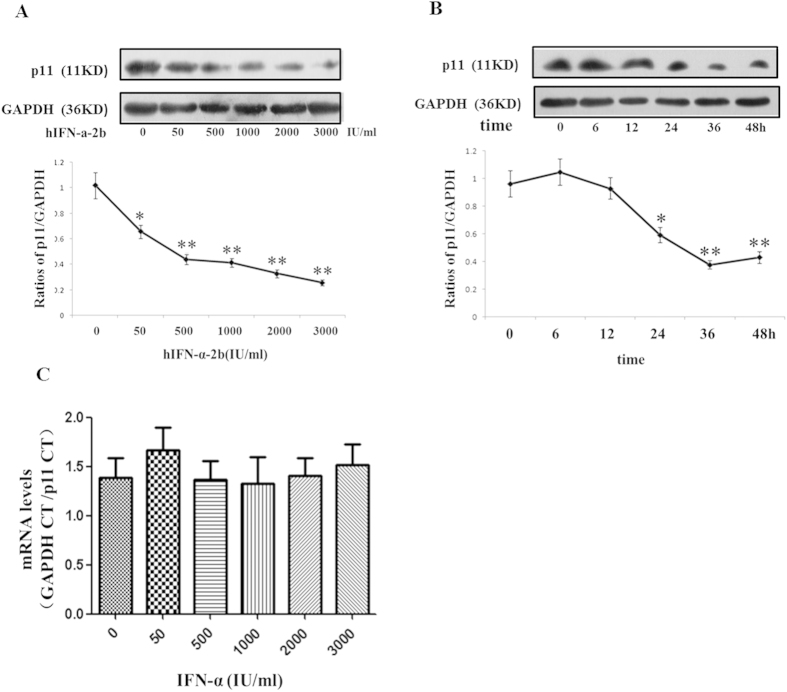
The p11 protein and mRNA levels in SH-sy5y cells treated with IFN-α. (**A,B**) The p11 protein levels were measured using western blots. The p11 protein levels were normalized against GAPDH. The results demonstrated that IFN-α treatment down-regulated the p11 protein levels in SH-sy5y cells. (**A**) Different doses of hIFN-α-2b (0, 50, 500, 1000, 2000, and 3000 IU/mL) for 24 h produced significant dose-dependent decreases in the p11 protein levels. (**B**) Time-dependent (0, 6, 12, 24, 36, and 48 h) effects in IFN-α (1000 IU/mL) treated cells, with reduced p11 protein levels at 24 h and the lowest levels at 36 h. (**C**) Dose-dependent effect of IFN-α on the p11 mRNA levels in SH-sy5y cells, which were normalized against GAPDH. SH-sy5y cells were treated with hIFN-α-2b (0, 50, 500, 1000, 2000, and 3000 IU/mL) for 16 h, and p11 mRNA levels were measured using real-time PCR. The real-time PCR results demonstrated no effects of hINF-α-2b on the p11 mRNA levels. All results were representative of three separate experiments. The data represented the mean ± S.E. compared with the controls. *P < 0.05 and **P < 0.01.

To investigate regulation effects of IFN-α on p11, SH-sy5y cells were treated with different dosages of IFN-α for various time-durations. The results in dose-dependent tests showed that hIFN-α-2b reduced the p11 protein levels in a dose-dependent manner (Fig. 4A). Compared with the controls (0 IU/mL of hIFN-α-2b), the p11 protein levels in the 50, 500, 1000, 2000, and 3000 IU/mL hIFN-α-2b groups were significantly reduced by 35.5% (*P* = 0.0408*, F* = *2.908*), 56.9% (*P* = *0.0068, F* = *5.848*), 59.3% (*P* = *0.0057, F* = *6.875*), 70.0% (*P* = *0.0033, F* = *8.684*), and 74.9% (*P* = *0.0022, F* = *10.4*), respectively. However, for p11 mRNA levels, no remarkable differences were observed between any concentrations of hIFN-α-2b groups and the controls (Fig. 4C).

The results in time-dependent tests suggested that the p11 protein levels were significantly decreased after hIFN-α-2b treatment at 24 (*P* = *0.0213, F* = *4.232*), 36 (*P* = *0.004, F* = *6.355*), and 48 h (*P* = 0.0059*, F* = *4.709*) compared with the pre-treatment p11 protein levels (Fig. 4B). Also, regression analyses showed significant negative correlations between protein levels and dosages of IFN-α (r = −0.849, *P* = 0.000) and between protein levels and durations of IFN-α treatment (r = −0.882 *P* = 0.000).

**Figure 5:**
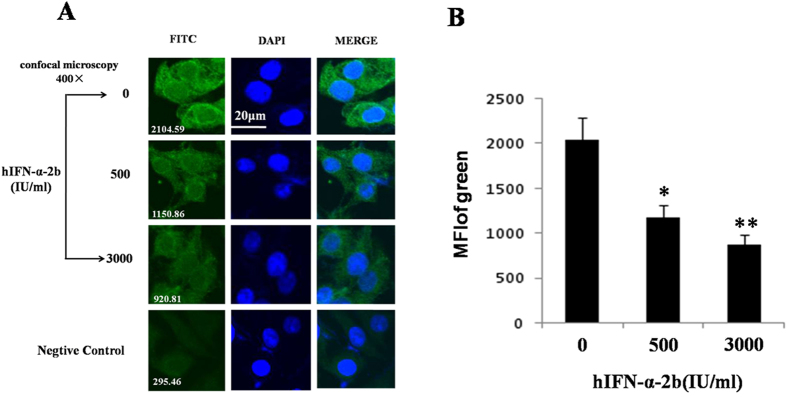
Immunofluorescence staining assayed the p11 protein in SH-sy5y cells treated with hIFN-α-2b (0, 500, and 3000 IU/mL) for 24 h. The cells were stained with antibodies against p11 (primary antibody) and FITC-labeled secondary antibodies. (**A**) The FITC column in the micrographs showed the p11 protein levels. The white numbers at the bottom left of each section represented the average fluorescent intensity (MFI). In the middle column, DAPI staining shows the cells nuclei. Merged images of FITC and DAPI staining were shown in the column on the right. Confocal microscopy was conducted at 400× and the scale bar represents 20 μm. The negative control group was stained with rabbit IgG instead of the primer antibody. (**B**) Histogram of the mean fluorescence intensity of FITC. All results were representative of three separate experiments. The data represented the mean ± S.E. compared with the controls. *P < 0.05 and **P < 0.01.

To confirm the down-regulation effects of IFN-α on p11, p11 levels in SH-sy5y cells were assayed with immunofluorescence. SH-sy5y cells were fixed and stained after treatment with different doses of hIFN-α-2b (0, 500, and 3000 IU/mL) for 24 h. Microscopic analysis indicated that mean fluorescence intensity (MFI) of the p11 protein in the 500 and 3000 IU/mL IFN-α treatment groups significantly declined by 41.9% (*P* = *0.0201, F* = *3.592*) and 56.7% (*P* = *0.0082, F* = *6.129*), respectively, compared with the controls (Fig. 5A,B).

### IFN-α remarkably decreased the 5-HT1Rb/4 protein levels in a time- and dose-dependent manner

**Figure 6:**
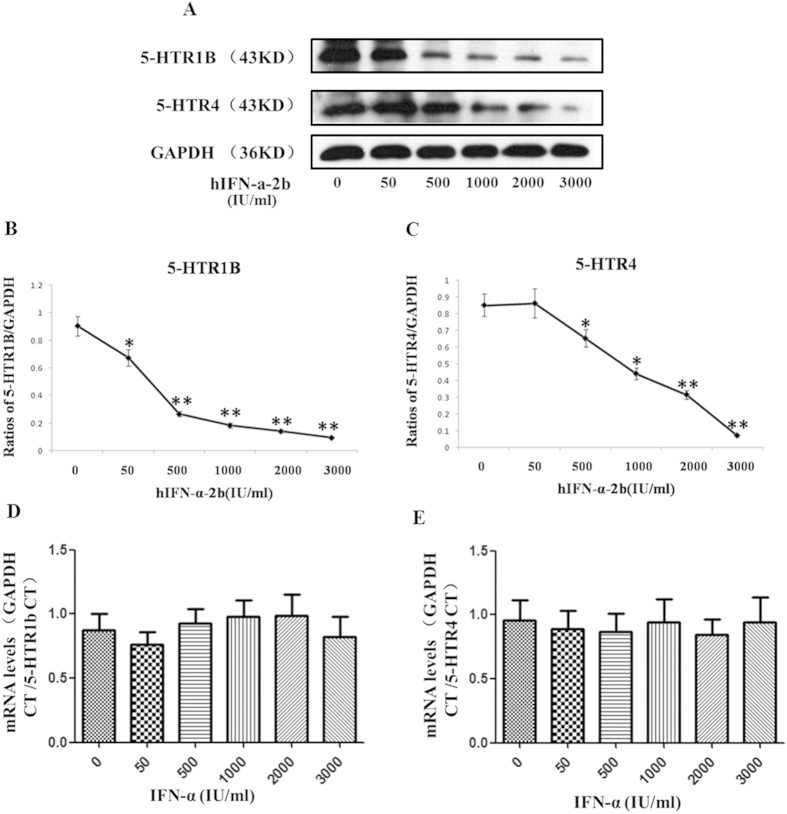
Regulation effects of IFN-α on protein and mRNA levels of 5-HTR1b/4 in SH-sy5y cells. (**A–C**) IFN-α treatment down-regulated the 5-HTR1b and 5-HTR4 protein levels in a dose-dependent manner. The 5-HTR1b and 5-HTR4 protein levels were determined using western blotting. Cells were treated with different doses of hIFN-α-2b (0, 50, 500, 1000, 2000, and 3000 IU/mL; 0 IU/mL as the control) for 24 h. (**A**) The western blots detected a significant dose-dependent decrease in the 5-HTR1b/4 protein levels after hIFN-α-2b treatment. The 5-HTR1b (B) and 5-HTR4 (**C**) protein levels were normalized against GAPDH. (**D,E**) The real-time PCR results show that IFN-α treatment has no influence on the 5-HTR1b/4 mRNA levels in SH-sy5y cells. Cells were treated with different doses of hIFN-α-2b for 16 h. The values were normalized against GAPDH. (**D**) The 5-HTR1b mRNA levels in hIFN-α-2b treatment groups had no significant difference compared with the controls (*P* > 0.05). (**E**) The 5-HTR4 mRNA levels in hIFN-α-2b treatment groups had no significant difference compared with the controls (*P* > 0.05). All results were representative of three separate experiments. The data represented the mean ± S.E. compared with the controls. *P < 0.05 and **P < 0.01.

SH-sy5y cells were treated with IFN-α, and 5-HTR1b/4 levels were measured with western blots. The results in dose-dependent tests showed that the 5-HTR1b and 5-HTR4 protein levels were all reduced in a dose-dependent manner after IFN-α treatment (Fig. 6A). After treatment with 50, 500, 1000, 2000, and 3000 IU/mL hIFN-α-2b, the protein levels were reduced by 25.6% (*P* = *0.0455, F* = *2.076*), 70.5% (*P* = *0.0009, F* = *6.622*), 79.6% (*P* = *0.0005, F* = *12.85*), 84.3% (*P* = *0.0004, F* = *22.35*), and 89.5% (*P* = *0.0003, F* = *23.32*) in 5-HTR1b (Fig. 6B), and by −1.6% (*P* = *0.9156, F* = *1.811*), 23.0% (*P* = *0.0407, F* = *2.373*), 47.8% (*0.0103, F* = *4.533*), 62.7% (*P* = *0.0032, F* = 10.75), and 91.6% (*P* = *0.0007, F* = *158.8*) in 5-HTR4, respectively (Fig. 6C). No significant differences in mRNA levels of 5-HTR1b (Fig. 6D) or 5-HTR4 (Fig. 6E) between hIFN-α-2b treatment groups and the controls were found. Regressions analysis demonstrated that the dosages of IFN-α was negatively correlated to the protein levels of 5-HTR1b (r = −0.892, *P* = 0.000) and 5-HTR4 (r = −0.952, *P* = 0.000), respectively.

**Figure 7:**
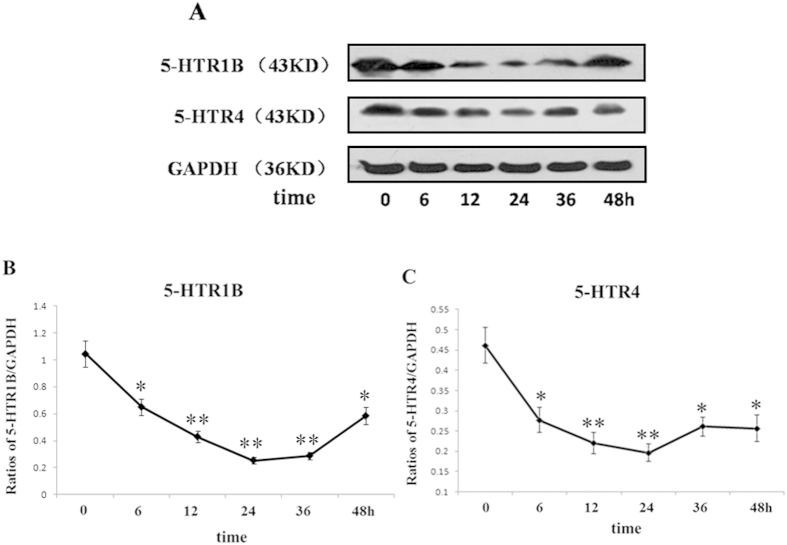
Changes in the 5-HTR1b and 5-HTR4 protein levels in SH-sy5y cells treated with IFN-α for different time periods. (**A**) Western blots showed the 5-HTR1b and 5-HTR4 protein levels in SH-sy5y cells treated with hIFN-α-2b (1000 IU/mL) at various time points (0, 6, 12, 24, 36, and 48 h). (**B,C**) The 5-HTR1b and 5-HTR4 protein levels were normalized against GAPDH. The results showed that the 5-HTR1b (**B**) and 5-HTR4 (**C**) levels declined at 6 h and reached their lowest levels at 24 h. All results were representative of three separate experiments. The data represented the mean ± S.E. compared with the controls. *P < 0.05 and **P < 0.01.

In the time-dependent tests, SH-sy5y cells were treated with 1000 IU/mL of hIFN-α-2b and collected at different time points (0, 6, 12, 24, 36, and 48 h). The 5-HTR1b and 5-HTR4 protein levels were determined using western blots (Fig. 7A). The results showed that the 5-HTR1b or 5-HTR4 protein levels at 12 h were significantly reduced by 24.4% (*P* = *0.0043, F* = *4.763*), and 39.9% (*P* = *0.0078, F* = *4.912*), respectively, compared with the controls. At 24 h, the 5-HTR1b and 5-HTR4 protein levels were reduced by 75.8% (*P* = *0.0013, F* = *15.27*) and 57.3% (*P* = *0.0051, F* = *11.79*), respectively, compared with the controls. The protein levels were all slightly higher at 48 h than at 24 h, but still were 44.1% (*P* = *0.0155, F* = *2.466*) for 5-HTR1b and 40.7% (*P* = *0.022, F* = *2.782*) for 5-HTR4 lower than the controls, respectively (Fig. 7B,C). Regressions analysis exhibited there were significant negative correlations between the protein levels of 5-HTR1b (r = −0.959, *P* = 0.000) and 5-HTR4 (r = −0.774, *P* = 0.000) with the time durations of IFN-α treatment.

### The protein levels of p11, 5-HTR1b and 5-HTR4 in the cytomembrane of SH-sy5y cells were significantly reduced after IFN-α treatment

**Figure 8:**
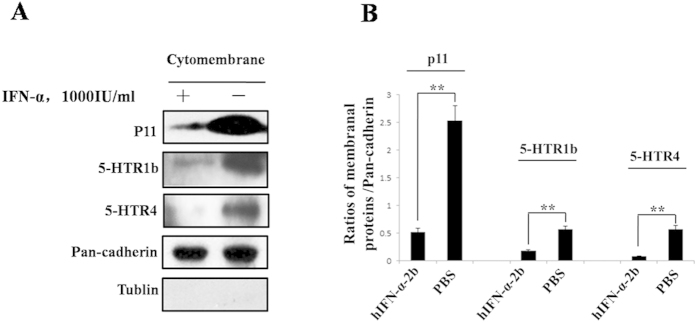
The 5-HTR1b and 5-HTR4 protein levels in cytomembrane of SH-sy5y cells after IFN-α treatment. (**A**) SH-sy5y cells were treated with 1000 IU/mL hIFNα-2b, and with an equal volume of PBS as the control. The proteins in cytomembrane of SH-sy5y cells were extracted at 24 h. The p11, 5-HTR1b and 5-HTR4 protein levels were analyzed using western blotting. (**B**) The levels of p11, 5-HTR1b and 5-HTR4 protein in cytomembrane were normalized against Pan-cadherin. Tublin test was performed to eliminate the contamination from cytoplasm to cytomembrane proteins. All results were representative of three separate experiments. The data represented the mean ± S.E. compared with the controls. **P < 0.01.

We further investigated the effects of IFN-α treatment on the protein levels of p11, 5-HTR1b and 5-HTR4 in the cytomembrane of SH-sy5y cells. SH-sy5y cells were treated with 1000 IU/mL of hIFN-α-2b or equal volume of PBS. At 24 h, the cells were collected, and then membranal proteins were extracted for western blot analysis to determine the p11 and 5-HTR1b/4 protein levels. The results showed that there were no tublin protein bands in membrane protein samples (Fig. 8A). It was indicated that there was no cross-contamination between membranal and cytoplasmic protein extracts. We found that the protein levels of p11, 5-HTR1b and 5-HTR4 in membrane were all dramatically decreased in IFN-α treatment groups compared with the controls (Fig. 8B). After IFN-α treatment, levels of p11, 5-HTR1b and 5-HTR4 protein in cytomembrane declined by 79.34% (*P* = *0.0014, F* = *19.45*), 68.42% (*P* = *0.0039, F* = *11.77*) and 85.31% (*P* = *0.0019, F* = *43.6*), respectively, compared with the controls.

### p11 controlled protein levles of 5-HTR1b and 5-HTR4, and over-expression of p11 prevented the IFN-α-induced down-regulation of 5-HTR1b and 5-HTR4

**Figure 9:**
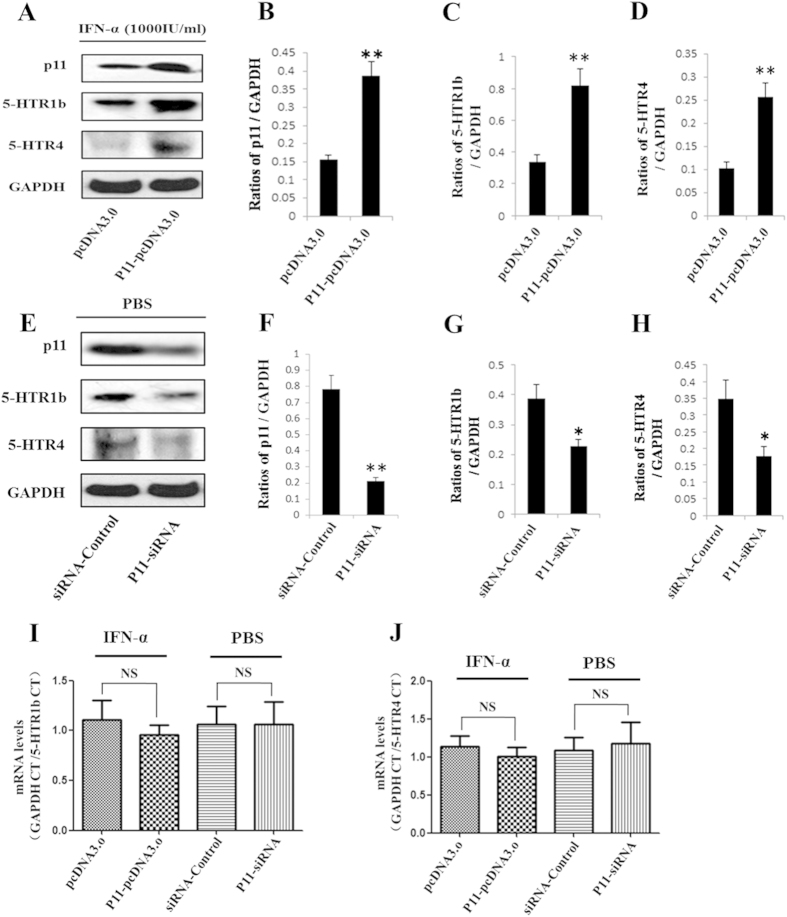
The 5-HTR1b and 5-HTR4 protein or mRNA levels in SH-sy5y cells transfected with p11-pcDNA3.0 after IFN-α treatment or with p11-miRNA vector in the absence of IFN-α. After plasmid transfection for 24 h, 1000 U/mL hIFNα-2b or PBS was added to the medium. Total proteins were extracted at 48 h. and the p11, 5-HTR1b and 5-HTR4 protein levels were analyzed using western blotting. Total RNA was extracted at 36 h and reverse transcription was applied to produce cDNA. The 5-HTR1b and 5-HTR4 mRNA levels were analyzed using real-time PCR. (**A**) SH-sy5y cells were transfected with p11-pcDNA3.0 containing full-length p11cDNA, with pcDNA3.0 as the control. (**B–D**) The p11 (**B**), 5-HTR1b (**C**), and 5-HTR4 (**D**) protein levels in p11-pcDNA3.0 transfection groups were normalized against GAPDH. (**E**) After PBS treatment, SH-sy5y cells were transfected with p11-miRNA that interfered with the expression of p11, miRNA-control vector as the control. (**F–H**) The p11 (**F**), 5-HTR1b (**G**), and 5-HTR4 (**H**) protein levels in p11-miRNA transfection groups were normalized against GAPDH. (**I,J**) mRNA levels of 5-HTR1b (**I**) and 5-HTR4 (**J**) in SH-sy5y cells in each transfection group was analyzed using real-time PCR and normalized against GAPDH. All results were representative of three separate experiments. The data represented the mean ± S.E. compared with the controls. *P < 0.05, **P < 0.01 and *NS*, no significant difference.

We found that IFN-α significantly reduced the protein levels of p11, 5-HTR1b and 5-HTR4 in *in vitro* and *in vivo* experiments. Here, we sought to determine whether p11 mediated the down-regulating effects of IFN-α on the levels of 5-HTR1b or 5-HTR4. Thus, we performed experiments to over-express or knockdown p11. Transfection with the p11-pcDNA3.0 plasmid generated high levels of full-length p11 (Fig. 9A), whereas transfection with the p11-miRNA vector inhibited the expression of the p11 protein (Fig. 9E). The p11 protein levels increased 150.5% (*P* = *0.0048, F* = *7.329*) in the p11-pcDNA3.0 groups (Fig. 9B) even though hIFN-α-2b was added. On the other hand p11 protein levels reduced by 72.8% (*P* = *0.0038, F* = *8.306*) in the p11-miRNA transfected groups, compared with the controls (Fig. 9F).

In parallel, similar changes in the 5-HTR1b and 5-HTR4 protein levels were observed in the p11-pcDNA3.0 groups or in the p11-miRNA groups. After hIFN-α-2b treatment, the 5-HTR1b or 5-HTR4 protein levels in the p11-pcDNA3.0 groups increased by 141.9% (*P* = *0.0081, F* = *4.324*) or 148.9% (*P* = *0.0064, F* = *4.618*), respectively, compared with the controls (Fig. 9C,D). On the contrary, even if in the absence of hIFN-α-2b, the 5-HTR1b or 5-HTR4 protein levels in the p11-miRNA groups were reduced by 41.6% (*P* = *0.0302, F* = *3.684*) or 49.3% (*P* = *0.0224, F* = *6.372*), respectively, compared with the controls (Fig. 9G,H). Such results indicated that 5-HTR1b and 5-HTR4 expressions were stringently correlated with p11, and more importantly over-expression of p11 was sufficient to prevent the IFN-α-induced inhibition on 5-HTR1b and 5-HTR4.

In addition, when mRNA levels of 5-HTR1b and 5-HTR4 were assayed using real-time PCR, the results showed that neither 5-HTR1b nor 5-HTR4 mRNA levels in the p11-pcDNA3.0 groups had significant difference compared with the controls (Fig. 9I). Similar results were observed in the p11-miRNA groups compared with the controls (Fig. 9J).

The statistical power analysis showed the powers are greater than 90% for each experiment of all, indicating that the number of subjects met the demand of statistic analysis.

## Discussion

It is not uncommon for IFN-α treatment to induce the onset of a severe mental disorder, particularly major depression^5^. The mechanisms underlying this process remain unclear, but several hypotheses suggest that IFN-α-induced depression may be related to the effects of IFN-α on tryptophan metabolism, the synthesis of 5-hydroxytryptamine, and/or the activity of serotonin reuptake receptors^6^,^31^,^32^. However, previous studies are still controversial in their explanations of the molecular mechanisms responsible for IFN-α-induced depression. It has been reported that the deficiency of p11 plays key roles in the onset of depression via impairing the functions of its downstream chaperonins 5-HTR1b/4^17^,^33^,^34^. In the present study, we therefore determined whether the deficiency of p11 was involved in IFN-α-induced depression, whether p11 altered the levels of 5-HTR1b and 5-HTR4, and whether the regulation effects of IFN-α on levels of 5-HTR1b and 5-HTR4 were dependent upon p11.

First of all, we assayed depression-like behavior in mice after IFN-α injection using forced swimming tests and tail suspension tests. We found treatment with IFN-α could increase the immobility of the Balb/c mice in FST and TST (Fig. 1A,B). The results indicated that IFN-α treatment could cause depression-like behavior in mice. Animals’ responses in physiology and behavioral psychology to IFN-α treatment are different, it may be due to the source of IFN-α, administration routes, the dosage, injection duration and genetic backgrounds. It is reported that, when administrated with murine IFN-α (60,000 IU/kg for 8 consecutive days, intraperitoneal injection), the mice exhibit subtle behavioral changes in the FST, but not TST^35^. hIFN-α-2b (400–1600 IU/g/day for 5–15 days, subcutaneously) significantly increases the immobility in the FST^36^. A more recent study finds that treatment with murine IFN-α (250 IU/day) for 14 days results in a reproducible depression-like state that can be characterized by increased immobility of C57BL6/J mice in TST and FST^37^. Therefore, we evaluated the depression-associated behavior of Balb/c mice that were treated with hIFN-α-2b (1000IU/g/day, intraperitoneal injection) for 10 days. In addition, in this study we chose human instead of murine IFN-α to treat mice, based on the consideration that clinical side effects were the result of human IFN-α treatment, though their functions in mice could be different^38^,^39^.

Studies have shown that p11, 5-HTR1b and 5-HTR4 proteins in certain areas of rodent brains (e.g., hippocampus^27^,^34^,^40^, cortex^21^,^41^, amygdala^21^,^42^ and nucleus raphe^29^) play key roles in the onset of depression. Using IHC, we evaluated these protein levels in mice brains after IFN-α treatment. We found that continuous intraperitoneal injections of IFN-α into Balb/c mice led to lower protein levels of p11, 5-HTR1b and 5-HTR4 in the hippocampus or cingulate gyrus (Fig. 2), and the results were further confirmed with western blots (Fig. 3). Both the hippocampus and cingulate gyrus belong to the limbic system, which is involved in emotion formation and processing, learning, and memory. The combination of these functions makes the hippocampus and cingulate gyrus highly influential in linking behavioral outcomes to motivation^43^,^44^, and the roles make them highly important in mental disorders, particularly depression^45^,^46^. The hippocampus and cingulate gyrus contain abundant serotonergic nerve fibers expressing 5-HTR1b and 5-HTR4^47^. It is found that 5-HTR1b and 5-HTR4 can co-localize with p11 in hippocampus and play crucial roles in anti-depression effects in mice treated with SSRIs^48^. Results from our study and previous ones indicated that lower levels of these proteins in hippocampus or cingulate gyrus could be critical in IFN-α-induced depression. In addition, the western blot data suggested some more dramatic hippocampal down-regulation of p11 and 5-HTR1b/4 protein levels by IFN-α treatment than the IHC quantification (Figs 2 and 3). This was probably due to the fact that western blotting was more sensitive than IHC in the proteins detection^49^.

Next, we investigated the effects of IFN-α on the expressions of p11 and 5-HTR1b/4 in SH-sy5y cell line. Our tests showed that the p11, 5-HTR1b and 5-HTR4 protein—but not mRNA—levels were significantly reduced in a dose- and time-dependent manner after treatment with IFN-α (Figs 4, 5, 6, 7). Furthermore, we also found that the levels of p11 and 5-HTR1b/4 proteins in cytomembrane were remarkably declined after IFN-α treatment (Fig. 8). Several studies have shown that 5-HTR1b and 5-HTR4 proteins play key roles in the anti-depression effects associated with p11^18^,^50^. Both 5-HTR1b and 5-HTR4 are G protein-coupled transmembrane receptors that produce different functions in response to 5-HT^51^. The functions in both the peripheral and central nervous systems are to modulate the release of various neurotransmitters^29^,^52^. It was not difficult to speculate that the decreased 5-HTR1b/4 protein levels in the membrane of neural synapses after IFN-α treatment would affect the functions in serotonergic neurons. We noted a recovery of protein levels for 5-HTR1b and 5-HTR4 at 48 h after IFN-α treatment (Fig. 7B,C), and such single dose curves shown in Fig. 7B,C were commonly seen in other researches^53^,^54^. Generally, a single dose treatment tended to affect the expression in protein level or mRNA level in a certain period of time and the effect would decrease over time. The protein levels of 5-HTR1b and 5-HTR4 should be fully recovered after the IFN-α was degraded. We did not test for a longer period of time because of the limitations of the cell culture procedure–the medium had to be changed after 48 h. On the other hand, the IFN-α is usually clinically administered on a daily basis^55^.

To investigate whether the down-regulating effects of IFN-α on 5-HTR1b or 5-HTR4 were mediated by p11, we analyzed 5-HTR1b or 5-HTR4 protein levels under the condition of higher or lower levels of p11 protein in SH-sy5y cells. We found that the 5-HTR1b and 5-HTR4 protein levels greatly increased in the p11-pcDNA3.0 transfection group (Fig. 9A–D), and they significantly declined in the p11-miRNA transfection group (Fig. 9E–H), compared with the controls. The data indicated that the 5-HTR1b and 5-HTR4 protein levels were positively correlated with the p11 protein levels even if treated with IFN-α. Such results indicated that the down-regulating effects of IFN-α on 5-HTR1b and 5-HTR4 were controlled by p11.

Although Svenningsson and Warner-Schmidt had reported that p11 can alter the distributions of 5-HTR1b/4 in cells by transferring them from the cytoplasm to the cytomembrane and thus increasing the density of 5-HTR1b/4 in the cytomembrane, it is still unknown whether p11 has the ability to modulate 5-HTR1b/4 protein levels^17^,^18^. Our studies suggested that p11 could affect 5-HTR1b/4 protein levels. In addition, we found decreased levels of p11 protein and depressive behaviors in mice treated with IFN-α, indicating a correlation between lower levels of p11 and depression-like behaviors.

Several studies have demonstrated that both 5-HTR1b and 5-HTR4 are critical for the initiation of depression. In the pre-synapses, 5-HTR1b acts as an autoreceptor to inhibit the release of serotonin^56^. Cai *et al.* demonstrate that the activation of 5-HTR1b in post-synapse facilitates excitatory synaptic transmission, which is associated with depression^25^. It has been suggested that 5-HT1b antagonists may be effective adjunctive therapies for depression^57^. 5-HTR4 was originally identified as a mediator of 5-HT. Further studies indicated that the activation of 5-HTR4 increases the adenylate cyclase activity in mouse colliculi neurons, which subsequently accelerates the recovery of rapid excitatory postsynaptic potentials from rundown^58^. 5-HTR4 enhances the release of a number of neurotransmitters, and 5-HTR4-knockout mice exhibit an exaggerated inhibitory response in the 5-HT neurons to the anti-depressant reagent citalopram^59^. These evidences demonstrated that lower levels of 5-HTR1b or 5-HTR4 protein might cause the onset of depression.

We found that IFN-α administration resulted in depression-like behavior in mice and inhibited the protein levels of p11, 5-HTR1b and 5-HTR4 in SH-sy5y cells and the brains of mice. Furthermore, our results also suggested that the reduction in the protein levels of 5-HTR1b and 5-HTR4 were dependent upon p11 after IFN-α treatment. Together, these results illustrated that p11, 5-HTR1b, and 5-HTR4 play key roles in IFN-α-induced depression. These receptors might be involved in the mechanism underlying the inhibitory effects of IFN-α on the protein levels of p11 in brain areas (e.g., hippocampus, cingulate gyrus, and other regions) related to depression. Lower p11 levels probably led to a decline in the protein levels of 5-HTR1b and 5-HTR4 in local nerve synapses, subsequently disturbing the transmission of neurotransmitters in the synapses, and ultimately causing the body to experience depression. Also, there might be other molecules besides p11 involved in IFN-α-induced depression, which we did not examine. Further experiments using p11 knockout mice might help elucidate this issue. If the mice developed depression deferentially after IFN-α injection compared to the wild-type mice, IFN-α-induced depression might involve additional mechanisms besides p11. It is noteworthy that only a subsection of the individuals become depressed following IFN-α treatment, which could be explained by the variation of genetic makeup of an individual^60^,^61^,^62^. Further studies would allow us to clarify the molecular mechanism of IFN-α-induced depression.

Furthermore, p11 can be a potential biomarker in depression, as studies suggest that p11 mRNA levels in peripheral blood mononuclear cells correlate with suicide risk in mental disorders^33^ and can distinguish post-traumatic stress disorder from bipolar disorder or major depression^63^. p11 is considered to be an up-stream regulator in the translocation and signal transduction of 5-HTR1b and 5-HTR4^17^,^18^,^29^. Our study found that IFN-α dramatically down-regulated p11 protein levels in a dose- and time-dependent manner, and that p11 controlled 5-HTR1b/4 protein levels. We suspected that p11 protein could be a potential biomarker in monitoring IFN-α-induced depression. In addition, we found IFN-α reduced p11 protein but not mRNA levels in cells, which were obviously different from the p11 mRNA studies in other types of depression^33^,^63^. Our findings illustrated that p11 protein—but not mRNA—levels could be a potential biomarker in IFN-α-induced depression, although clinical investigation is needed

## Methods

### Animals and drug treatments

The Balb/c mice were purchased from Vital River Laboratory Animal Technology Company (Beijing), and housed in specific-pathogen-free conditions. This study was approved by and performed in accordance with guidelines established by the Xinxiang Medical University Committee on the Use and Care of Animals. Adult male wild-type Balb/c mice were used in the animal tests. All mice were maintained with a 12-h light/dark cycle, and food and water were provided ad libitum. For drug treatments, three or four mice were housed per cage and hIFN-α-2b was injected into mice for 10 uninterrupted days (1000 IU/g/day, intraperitoneal injection, total volume = 0.2 mL)^36^. hIFN-α-2b was purchased from Sigma and diluted with PBS. Mice were injected with equal volumes and frequencies of PBS as the controls. The mice were injected at 9 a.m. each day.

### Mice behavior tests

FST were carried out 1–2 days after the last IFN-α treatment on mice according to the method described in literature^64^. A video tracking system (Yishu, China) equipped with two 4 × 4 photo beam arrays allowing for the monitoring of swimming behaviors was used. Two swim sessions were conducted: the day before the start of the test, the mice were placed into the water to acclimatize them to FST. For testing, mice were individually and gently placed in a glass cylinder (30 cm high, 10 cm in diameter) filled with water at 24–26 °C to a depth of 18 cm, and allowed to swim for 6 min. The data of immobility within 2–6 min were collected to measure the behaviors.

TST was performed one day after the last IFN-α treatment on mice. Briefly, mice were suspended in the testing equipment (Bioseb, USA) by taping the distal part of the tail (1–1.5 cm) to a flat metallic surface 40 cm above the floor. Escape movements were recorded for 6 min. The time spent in an immobile posture within 2–6 min was measured as an index of depression-like behavior.

### Immunohistochemistry

Fifty hours after the last IFN-α injection, the mice were anesthetized with ether. The brains were perfused with 4% ice-cold paraformaldehyde and treated with a series of processes to achieve dehydration, clearing, and wax immersion. The tissues were cut in a coronal plane for 5 μm-thick sections, which included areas of the hippocampus and cingulate gyrus. The sections were subjected to a series of processes for dewaxing, rehydration, inactivation of endogenous enzymes (immersion in 3% H_2_O_2_ for 30 min at room temperature), and antigen repair (immersion in citrate buffer at 99 °C for 30 min, followed by cooling to room temperature). The immune staining steps used streptavidin-biotin complex (SABC) immunohistochemistry kits (Biosail, China). First, the sections were blocked with 5% BSA for 30 min at 37 °C, followed by incubation with the primary antibody (anti-p11 1:300, Proteintech; anti-5-HTR1b 1:500, Abcam; anti-5-HTR4 1:500, Abcam; 1/1000 rabbit IgG as a control, Biosail, China). Second, after three washes with PBS, the sections were incubated with the secondary antibody (biotinylated goat anti-rabbit IgG, 1:200) at 37 °C for 30 min. Third, after three washes with PBS, the sections were incubated with 1/100 SABC reagents (Biosail, China) at 37 °C for 30 min. Fourth, the sections were stained with hematoxylin (Solarbio, USA) using a DAB chromogenic kit (Bostail, China). Finally, the sections were dried, sealed with a slide cover, and observed using an optical microscope (Olympus BX53, Japan).

### Preparation of hippocampus proteins

Fifty hours after the last IFN-α injection, 5 mice in each group were anesthetized with ether, and the brains were removed and dissected on ice-cold PBS. Hippocampi were removed and pulverized completely in liquid nitrogen. The samples were mixed with 10 folds volume (μL/μg) of lysis buffer (Beyotime, Beijing) that contained 1% Triton X-100, 1% deoxycholate, and 0.1% sodium dodecyl sulfate (SDS), and supplemented with protease inhibitor cocktail (Abcam, USA). After being centrifuged at 12,000 rpm for 5 min at 4 °C, the supernatants were collected and stored at −80 °C for western blotting analysis.

### Cell culture

Human neuroblastoma cell line (SH-sy5y cell line, Jiahe Biotech Company, Shanghai, China) was cultured in Dulbecco’s modified Eagle medium (DMEM) containing 10% fetal bovine serum, 50 U/mL penicillin, and 50 mg/mL streptomycin at 37 °C, with 5% CO_2_. All of the experiments were performed when the cells were 80–90% confluent.

### hIFN-α-2b treatment

#### Dose-dependent test

Cells were refreshed with DMEM and treated with a series of hIFN-α-2b (0, 50, 500, 1000, 2000, and 3000 IU/mL) as reported^65^. The cells were harvested at 16 h for mRNA measurement, and at 24 h for protein detection and immunofluorescence staining.

#### Time-dependent test

Cells were treated with a single dose of 1000 IU/mL hIFN-α-2b and harvested at 0, 6, 12, 24, 36, and 48 h for protein detection.

### Plasmid construction

To construct p11 over-expression plasmid, a 319-bp product of p11 cDNA was amplified using PCR. The forward primer was 5′-CCCAAGCTTATGCCATCTCAAATGG-3′ (with a *Hind*III restriction enzyme site), and the reverse primer was 5′-CGGAATTCCTACTTCTTTCCCTTC-3′ (with an *Eco*RI restriction enzyme site). The PCR products were cut using enzymes *Hind*III and *Eco*RI, followed by cloning into the pcDNA3.0 vector (Invitrogen, USA). The p11-miRNA plasmid (sense sequence: 5′-TGACACCTGAGAACTCATGGAAA-3′ and anti-sense sequence: 5′-TTTCCATGAGTACTCTCAGGT-3′) and its corresponding control plasmid (miRNA-control) (sense sequence: 5′-TGACGTCTCCACGCAGTACATTT-3′ and anti-sense sequence: 5′-AAATGTACTGCGCGTGGAGAC-3′) were constructed using pcDNA6.2-GW/EmGFP-miR vector by Invitrogen.

### Plasmid transfection and drug treatment

SH-sy5y cells were seeded at a density of 3 × 10^5^ cells per well in six-well plates and incubated in DMEM at 37 °C for 24 h. Before transfection, the cells were washed with antibiotic-free DMEM containing 10% fetal bovine serum. The plasmids were transfected into cells with Liposome Transfast^2000^ (Invitrogen, USA). The transfected plasmids contained p11-pcDNA3.0, pcDNA3.0 (control), P11-miRNA, and miRNA-control (control). At 5 h after transfection, the cells were washed with PBS and supplemented with complete DMEM. After incubation for another 24 h, 1000 IU/mL of hIFNα-2b or PBS was added to the medium. The cells were collected at 48 h and the total proteins were extracted using RIPA reagents (Beyotime, China).

### Cell membrane proteins extraction

SH-sy5y cells were treated with 1000IU/mL hIFN-α-2b and harvested at 24 h. Cytomembrane proteins were extracted with the Mem-PER eukaryotic membrane protein extraction kit (Thermo Fisher Scientific, USA) according to the manufacturer’s recommendations^66^. Anti-pan-cadherin (Abcam, USA) and anti-tublin (Abcam, USA) antibodies were applied to test the levels and the purity of membranal proteins^67^.

### Western blots

#### Sample preparation and electrophoresis

Cells were collected with trypsin (EPET, Biofluids) at specific time points. After three washes with cold PBS, the cells were re-suspended in RIPA lysis buffer, which contained 1% triton X-100, 1% deoxycholate, and 0.1% SDS (Beyotime, Beijing). The samples were vortexed and kept on ice for 30 min, followed by centrifugation (12000 rpm, 4 °C) for 5 min. The supernatants were collected in fresh tubes. The protein concentrations was measured using a BCA assay (Pierce, USA) according to the manufacturer’s instructions^68^.

#### Membrane staining with antibodies and film exposure

The PVDF membranes were blocked by incubation with 5% skim milk for 2 h at room temperature, followed by overnight incubation with the primary antibody at 4 °C (anti-p11 1:1000, Proteintech; anti-5-HTR1b 1:1000, Abcam; anti-5-HTR4 1:1000, Abcam and anti-GAPDH 1:2000, Santa Cruz). After washing with TBS-T (0.5% Tween 20, Sigma), the membranes were incubated with HRP-conjugated secondary antibodies for 1.5 h at room temperature (goat anti-rabbit 1:3000, Santa Cruz; goat anti-mouse 1:3000, Santa Cruz). The blot was developed using the ECL system (Beyotime, China) and exposed to a radiographic film. The gray scales of the bands were quantified using “Quantity-One” software.

### Immunofluorescence

The cells were plated onto sheet glasses for the immunofluorescence staining tests. At specific time points, the cells were washed with cold BSA-TBS (1 g/L BSA, 2.42 g/L Tris-base, 8 g/L NaCl, pH 7.6) and then fixed using −20 °C pre-cooled 70% ethanol at room temperature for 10 min. The sections were washed twice with TBS-T (0.25% Triton X-100, Sigma) for 15 min each time. Subsequently, the samples were blocked using 5% BSA (diluted with TBS-T) for 1 h at room temperature and then incubated overnight with the primary antibody (anti-p11 1:300, Proteintech; 1/1000 rabbit IgG as negative control, Beyotime, China) at 4 °C. After five washes with TBS-T, the sections were incubated with an FITC-labeled secondary antibody (goat anti-rabbit IgG-FITC 1:2000, Santa Cruz) for 1 h at room temperature, followed by three washes with TBS-T. Next, the cells were stained with DAPI (4,6-diamidino-2-phenylindole, Invitrogen) for 20 min and sealed with 20 μL antifade mounting medium. Finally, the sections were observed using a laser confocal microscope (Olympus FV1000, Japan).

### RNA extraction and real-time PCR

Cells were collected at 16 h and the total RNA was extracted using an RNA extraction kit (Minico, USA). One microgram of total RNA was used to synthesize the first-strand cDNA using the Superscript III reverse transcription kit (Invitrogen, USA). The cDNA was assayed using real-time PCR with a GoTaq kit (Promega, USA) the manufacturer’s instructions^69^. The real-time PCR primers were obtained from Biosail: the p11 forward primer was 5′-AAATTCGCTGGGGATAAAGG-3′ and reverse primer was 5′-AGCCCACTTTGCCATCTCTA-3′. The 5-HTR4 forward primer was 5′-TTATGGGGAGGTGTTTTGTCTT-3′ and reverse primer was 5′-GCAGAGGGGTCATCTTGTTC-3′. The 5-HTR1b forward primer was 5′-AACAAGTCAAAGTGCGAGTCT-3′ and reverse primer was 5′-GGAGATGATGAAGAAGGGTAGC-3′. The GAPDH forward primer was 5′-TCAACAGCGACACCCACTCC-3′and reverse primer was 5′-TGAGGTCCACCACCCTGTTG-3′.

### Statistical analysis

The *P* values in all experiments were determined with Prism software (GraphPad Software Inc.) for nonparametric unpaired T tests. F tests were used to compare variances. Regression analyses were performed with SPSS19.0. We considered *P*-values < 0.05 to be significant and the degree of significance is indicated as follows: *P < 0.05, **P < 0.01. In addition, to ensure the number of subjects meet the demand of statistics, the statistic power was calculated (alpha = 0.05, two tails).

## Additional Information

**How to cite this article**: Guo, J. *et al.* Probable involvement of p11 with interferon alpha induced depression. *Sci. Rep.* **6**, 17029; doi: 10.1038/srep17029 (2016).
